# A randomized phase III study comparing dacarbazine, BCNU, cisplatin and tamoxifen with dacarbazine and interferon in advanced melanoma

**DOI:** 10.1054/bjoc.1999.1056

**Published:** 2000-02-21

**Authors:** M R Middleton, P Lorigan, J Owen, L Ashcroft, S M Lee, P Harper, N Thatcher

**Affiliations:** CRC Department of Medical Oncology, Christie Hospital NHS Trust, Wilmslow Road, Manchester, M20 4BX, UK; Department of Clinical Oncology, Weston Park Hospital, Witham Road, Sheffield, S10 2SJ, UK; Department of Medical Oncology, Guy's Hospital, St Thomas Street, London, SE1 9RT, UK; Meyerstein Institute of Oncology, Middlesex Hospital, Mortimer Street, London, W1N 8AA, UK

**Keywords:** melanoma, chemotherapy

## Abstract

The purpose of this study was to compare the response rate, overall and 1-year survival in patients with advanced melanoma treated with a standard therapy, dacarbazine and interferon-alpha (DTIC/IFN), or combination chemotherapy, consisting of dacarbazine, BCNU, cisplatin and tamoxifen (DBCT). Treatment toxicity and time spent in hospital were secondary end points. One hundred and five patients (of whom 100 were eligible) were randomized to receive either DTIC/IFN or DBCT. The trial was designed to detect a 25% absolute difference in response rate or in 1-year survival with 80% power. There was no significant difference in response rate: this was 17.3% with DTIC/IFN and 26.4% with DBCT. Median overall survival was similar at 199 and 202 days respectively. One-year survival rate favoured standard treatment (30.6 vs 22.6%), but did not differ significantly between arms. DBCT was associated with significantly greater haematological toxicity, and a greater need for time spent in hospital (5.75 days/treatment cycle vs 2.29 with dacarbazine and interferon). DBCT combination therapy cannot be recommended as standard treatment for advanced melanoma. Dacarbazine remains the standard chemotherapy for this condition. © 2000 Cancer Research Campaign


British Journal of Cancer (2000) **82**(6), 1158–1162
© 2000 Cancer Research Campaign
Article no. bjoc.1999.1056, available online at http://www.idealibrary.com on IDEAL®

### A randomized phase III study comparing dacarbazine, BCNU, cisplatin and tamoxifen with dacarbazine and interferon in advanced melanoma

**MR Middleton[1], P Lorigan[2], J Owen[2], L Ashcroft[1], SM Lee[4], P Harper[3] and N Thatcher[1]**

[1]CRC Department of Medical Oncology, Christie Hospital NHS Trust, Wilmslow Road, Manchester M20 4BX, UK; [2]Department of Clinical Oncology, Weston Park Hospital, Witham Road, Sheffield S10 2SJ, UK; [3]Department of Medical Oncology, Guy's Hospital, St Thomas Street, London SE1 9RT, UK; [4]Meyerstein Institute of Oncology, Middlesex Hospital, Mortimer Street, London W1N 8AA, UK

**Summary** The purpose of this study was to compare the response rate, overall and 1-year survival in patients with advanced melanoma treated with a standard therapy, dacarbazine and interferon-alpha (DTIC/IFN), or combination chemotherapy, consisting of dacarbazine, BCNU, cisplatin and tamoxifen (DBCT). Treatment toxicity and time spent in hospital were secondary end points. One hundred and five patients (of whom 100 were eligible) were randomized to receive either DTIC/IFN or DBCT. The trial was designed to detect a 25% absolute difference in response rate or in 1-year survival with 80% power. There was no significant difference in response rate: this was 17.3% with DTIC/IFN and 26.4% with DBCT. Median overall survival was similar at 199 and 202 days respectively. One-year survival rate favoured standard treatment (30.6 vs 22.6%), but did not differ significantly between arms. DBCT was associated with significantly greater haematological toxicity, and a greater need for time spent in hospital (5.75 days/treatment cycle vs 2.29 with dacarbazine and interferon). DBCT combination therapy cannot be recommended as standard treatment for advanced melanoma. Dacarbazine remains the standard chemotherapy for this condition. © 2000 Cancer Research Campaign

**Keywords:** melanoma; chemotherapy

The incidence of melanoma has increased over the last 40 years, more so than for any other tumour. It is now responsible for 7500 deaths annually in the USA. Although there have recently been interesting developments in the adjuvant treatment of surgically resected high-risk disease, the outlook for patients with advanced unresectable melanoma remains dismal. A minority of patients respond to treatment, remissions are generally only short-lived, and median overall survival is around 6 months (Balch et al, 1997). For many years dacarbazine has been the standard chemotherapy for melanoma, despite a response rate of only 20% and no discernible impact on survival (Lee et al, 1995). The relative ineffectiveness of single-agent treatment has led to considerable interest in multi-agent chemotherapy regimens, and in combining chemo- and biotherapy.

In 1984, del Prete reported the results of 20 patients treated with dacarbazine, BCNU, cisplatin and tamoxifen (DBCT). This four-drug combination, also known as the Dartmouth regimen, has since been at the forefront of attempts to improve response rates to chemotherapy and overall survival in patients with advanced melanoma. Although early studies observed response rates in excess of 50%, these have generally been in small numbers of patients (del Prete et al, 1984; McClay et al, 1992). To date no significant impact on overall survival has been reported, but long-term remissions have been described in some responders. However, until recently no randomized study had been published comparing DBCT with single-agent dacarbazine chemotherapy. Despite this, many centres have adopted DBCT as their standard therapy in advanced melanoma.

We designed a randomized phase III study to examine response rate and survival in patients treated with DBCT in comparison with a standard treatment of dacarbazine and interferon. We elected to use the latter regimen as our standard therapy as, at the time the trial was designed, there were reports to suggest that it was more effective than dacarbazine alone (Falkson et al, 1991). Improvements in survival, if seen, would need to be offset against the anticipated increase in toxicity and the need for hospitalization with multi-agent treatment. Thus, we decided to record the impact of DBCT on time spent in hospital during treatment, to help determine any extra costs associated with using DBCT.

### PATIENTS AND METHODS

### Patients

Adults (aged 18 years or more) with measurable advanced malignant melanoma were eligible for inclusion. Adequate performance status (Karnofsky score [KP] 50 or greater) and renal (creatinine clearance > 50 ml min$^{-1}$), hepatic (total bilirubin < 1.5 × the upper limit of laboratory normal [ULN], aspartate aminotransferase [AST] < 3 × ULN, alkaline phosphatase ≤ 3 × ULN), and bone marrow (absolute neutrophil count [ANC] ≥ 1500 mm$^{-3}$, platelets ≥ 100 000 mm$^{-3}$, haemoglobin ≥ 10 g dl$^{-1}$) functions were required. Prior therapy for metastatic melanoma was not permitted, with the exception of isolated limb perfusion or local radiotherapy for symptom control – so long as there was measurable disease beyond the treatment area. Patients with overt central

*Received 8 June 1999*
*Revised 11 September 1999*
*Accepted 23 September 1999*

*Correspondence to:* MR Middleton nervous system metastasis, pregnancy, or indications of a poor medical risk were excluded from the study. Individuals who had not recovered from previous treatment or who suffered from previous or concurrent malignancies at other sites were also excluded. Local ethical review committees approved the study. All patients gave written informed consent prior to randomization.

### Treatment

Patients were randomized through the Statistics Department at the Christie Hospital to receive either dacarbazine/interferon (DTIC/IFN) or DBCT. In the control arm, dacarbazine was administered intravenously (i.v.) on day 1 at a dose of 800 mg $m^{-2}$, and treatment was repeated every 21 days. IFN-α2a was given by subcutaneous injection at 9 Miu thrice weekly throughout the treatment period. Dose delay, not reduction, was practised when blood counts had not recovered at the time of planned re-treatment with dacarbazine. This was permitted when ANC > 1500 $mm^{-3}$ and platelets > 100 000 $mm^{-3}$. For the DBCT regimen dacarbazine and cisplatin were administered i.v. on days 1–3 at daily doses of 220 and 25 mg $m^{-2}$ respectively, with treatment being repeated every 21 days. BCNU was given i.v. at a dose of 150 mg $m^{-2}$ on day 1 and repeated every second cycle (i.e. every 42 days). Tamoxifen 20 mg was administered daily by mouth throughout treatment. Again, dose delay was practised when blood counts had not recovered at the time of planned re-treatment, with the same criteria as for DTIC/IFN. Treatment continued until unacceptable toxicity (as determined by individual investigators) or disease progression occurred, or for a maximum of six cycles. Both treatments were delivered in hospital, with DTIC/IFN generally requiring a 1-day admission and DBCT a 2-day stay.

### Study evaluations

Radiological and clinical evaluation of measurable sites of disease was required prior to randomization and after cycles 3 and 6. Where lesions were measurable on chest X-ray or abdominal ultrasound scan computerized tomography was not mandated. Responses were evaluated according to standard World Health Organization criteria. Toxicity was assessed and graded each cycle according to the Common Toxicity Criteria. In addition, the time spent in hospital was noted for each patient, without differentiating between admissions due to disease-related problems, treatment administration or toxicities.

**Table 1** Patient characteristics

| | | Dacarbazine/interferon *n* = 52 (%) | DBCT *n* = 53 (%) |
|---|---|---|---|
| Age | Median | 51 | 51 |
| | Range | 24–71 | 24–68 |
| Gender | Male | 31 (60) | 30 (57) |
| | Female | 21 (40) | 23 (43) |
| Initial KP | 50 | 1 (2) | 3 (6) |
| | 60 | 3 (6) | 4 (8) |
| | 70 | 8 (15) | 8 (15) |
| | 80 | 16 (31) | 14 (26) |
| | 90 | 12 (23) | 15 (28) |
| | 100 | 12 (23) | 9 (17) |
| Prior adjuvant interferon therapy | | 1 (2) | 4 (8) |
| Disease extent | No disease evident[a] | 2 (4) | 2 (4) |
| | Soft tissue/LN only | 15 (29) | 13 (25) |
| | Lung±soft tissue/LN | 9 (17) | 6 (11) |
| | Visceral[b] | 26 (50) | 32 (60) |

[a]These patients were not eligible (see text). [b]Excluding lung as the only visceral site.

### Statistical considerations

The primary objectives of the study were to compare response rate and 1-year survival between patients treated with either dacarbazine/interferon or DBCT, on an intention-to-treat basis. Survival was measured from the date of randomization to the date of death or last follow-up. Secondary objectives were to assess the median overall survival, toxicity and time spent in hospital for the two treatments. Recruitment of 100 patients was planned, to allow detection of a 25% absolute difference in survival at 1 year between arms in a two-tailed test, and a 25% difference in response rate with 80% power. Kaplan–Meier plots of survival were constructed, and these were compared using the log-rank test. Toxicities in each arm were evaluated by the $\chi^2$ test, and differences in hospital stay were assessed using a two-tailed Mann–Whitney *U*-test.

### RESULTS

Between 15 September 1994 and 15 June 1998, 105 patients were enrolled in the study, with 52 assigned to DTIC/IFN and 53 to DBCT chemotherapy. Five patients were not eligible for the study: three did not have metastatic melanoma, one had a prior malignancy and one lacked histological confirmation of melanoma. Three further patients did not receive treatment, two withdrew their consent after randomization, and one deteriorated rapidly and thus did not start therapy. These eight ineligible and/or untreated patients were evenly split between the two arms. Patient characteristics were well balanced between the two groups for factors known to affect response to therapy and survival (Table 1).

### Treatment

Five of the 105 randomized patients received no treatment, comprising two of the five ineligible patients, and the three who withdrew consent or deteriorated as described above. Fifty patients received dacarbazine and IFN, for a median four cycles. Fifty patients were treated with DBCT, also for a median of four cycles. Sixty-four of the 178 DBCT cycles given were delayed for toxicity, this was significantly more than with DTIC/IFN, where 22 of 194 cycles delivered were delayed ($P < 0.01$).

**Table 2** Response to treatment

| | Dacarbazine/interferon *n* = 52 (%) | DBCT *n* = 53 (%) |
|---|---|---|
| Ineligible and untreated | 3 (5.8) | 4 (7.5) |
| Not evaluable | 3 (5.8) | 2 (3.8) |
| Progressive disease | 32 (61.5) | 31 (58.5) |
| Stable disease | 5 (9.6) | 2 (3.8) |
| Partial response | 5 (9.6) | 12 (22.6) |
| Complete response | 4 (7.7) | 2 (3.8) |
| Overall response rate | 17.3% | 26.4% |

*© 2000 Cancer Research Campaign*

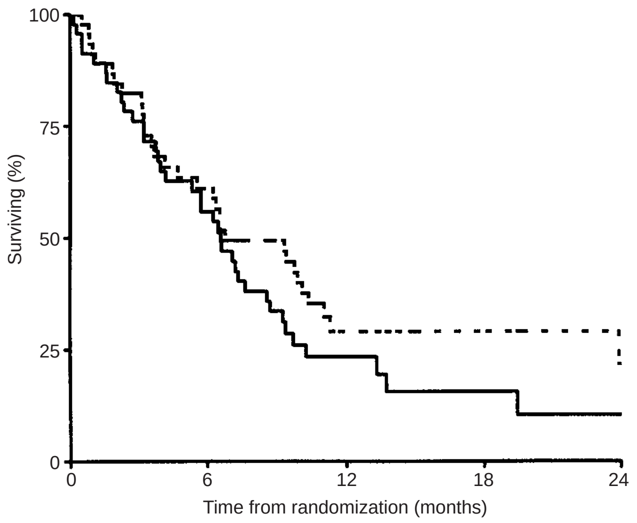

**Figure 1** Overall survival in patients treated with dacarbazine and interferon (------) or with DBCT (——)

### Response and survival

Response to treatment did not differ significantly between treatment arms, being 17.3% with DTIC/IFN (95% confidence interval (CI) 8.76–32.0%) and 26.4% with DBCT (95% CI 15.3–40.3%) in the intention-to-treat population ($P$ = 0.28; Table 2). Median survival was similar at 199 days with dacarbazine and IFN and 202 days with DBCT ($P$ = 0.39; Figure 1). The 1-year survival was superior for DTIC/IFN at 30.6% (95% CI 18.5–43.5%) compared with 22.6% (95% CI 12.8–34.1%) for DBCT, but this was not statistically significant ($P$ = 0.39). Amongst evaluable patients in the treated eligible population response rates were 19.6% (DTIC/IFN) and 29.8% (DBCT) and, again, were not significantly different. Responses to standard treatment lasted longer than with combination therapy (median 394 vs 247 days) but the difference did not achieve statistical significance ($P$ = 0.07).

### Toxicity

Data was available on the toxicity due to treatment for 96 of the 100 treated patients. Two patients were lost to follow-up, and two others died early of their melanoma before toxicity could be assessed. In all there were ten deaths on treatment, eight of which (four in each arm) were ascribed to progressive melanoma. There were two treatment-related deaths, both with DBCT: one patient died of sepsis when neutropenic, and another from intra-pulmonary haemorrhage whilst thrombocytopenic. Haematological toxicity was significantly greater with combination therapy: six of 50 patients on standard treatment had grade III or IV toxicity compared with 35 of 46 patients on DBCT. There were significant differences in the incidence and severity of anaemia, leucopenia and thrombocytopenia (Table 3). In addition, seven episodes of neutropenic fever requiring intravenous antibiotics were noted in six patients receiving DBCT, but there was only one such episode with DTIC/IFN. Otherwise, the treatments were well tolerated and although there was greater non-haematological toxicity with combination therapy this was only significant for increase in serum creatinine (Table 4).

**Table 3** Haematological toxicity

| | **Dacarbazine/interferon** ***n* = 50 (%)** | | | | | **DBCT** ***n* = 46 (%)** | | | | |
|---|---|---|---|---|---|---|---|---|---|---|
| Maximum grade of toxicity | 0 | 1 | 2 | 3 | 4 | 0 | 1 | 2 | 3 | 4 |
| Anaemia[a] | 32 (64) | 11 (22) | 5 (10) | 1 (2) | 1 (2) | 5 (11) | 9 (20) | 21 (46) | 10 (22) | 1 (2) |
| Leucopenia[a] | 27 (54) | 12 (24) | 6 (12) | 3 (6) | 2 (4) | 6 (13) | 4 (9) | 12 (26) | 17 (37) | 7 (15) |
| Thrombocytopenia[a] | 41 (82) | 4 (8) | 1 (2) | 2 (4) | 2 (4) | 5 (11) | 5 (11) | 5 (11) | 11 (24) | 20 (43) |

[a]$P < 0.001$; $\chi^2$ test.

**Table 4** Non-haematological toxicity

| | **Dacarbazine/interferon** ***n* = 50 (%)** | | | | | **DBCT** ***n* = 46 (%)** | | | | |
|---|---|---|---|---|---|---|---|---|---|---|
| Maximum grade of toxicity | 0 | 1 | 2 | 3 | 4 | 0 | 1 | 2 | 3 | 4 |
| Nausea and vomiting | 17 (34) | 17 (34) | 12 (24) | 4 (8) | | 11 (24) | 11 (24) | 16 (35) | 7 (15) | 1 (2) |
| Diarrhoea | 39 (78) | 10 (20) | 1 (2) | | | 42 (91) | 2 (4) | 2 (4) | | |
| Breathlessness | 39 (78) | 8 (16) | 2 (4) | | 1 (2) | 35 (76) | 4 (9) | 4 (9) | 2 (4) | 1 (2) |
| Stomatitis | 43 (86) | 7 (14) | | | | 40 (87) | 4 (9) | 2 (4) | | |
| Alopecia | 42 (84) | 5 (10) | 1 (2) | 2 (4) | | 31 (67) | 9 (20) | 4 (9) | 2 (4) | |
| Infection | 43 (86) | 4 (8) | 2 (4) | 1 (2) | | 34 (74) | 2 (4) | 4 (9) | 5 (11) | 1 (2) |
| Creatinaemia[a] | 49 (98) | 1 (2) | | | | 33 (72) | 10 (22) | 2 (4) | 1 (2) | |
| Bilirubinaemia | 46 (92) | 3 (6) | 1 (2) | | | 42 (91) | 2 (4) | | 2 (4) | |
| Transaminase rise | 33 (66) | 12 (24) | 4 (8) | 1 (2) | | 32 (70) | 7 (15) | 3 (7) | 3 (7) | 1 (2) |
| Alkaline phosphataemia | 37 (74) | 5 (10) | 4 (8) | 4 (8) | | 36 (78) | 6 (13) | 2 (4) | 2 (4) | |

[a]$P = 0.004$; $\chi^2$ test.

 *© 2000 Cancer Research Campaign*

### Hospitalization

Administration of DBCT required more time spent in hospital than with standard treatment (on average 2.0 days vs 0.94 days per cycle), but patients on combination therapy also spent more time in hospital for reasons other than receiving chemotherapy. On average DBCT patients spent 3.46 more days per treatment cycle in hospital (5.75 vs 2.29 days per cycle). When adjusted to account for time required for treatment delivery, the difference was 2.4 days per cycle (3.75 with DBCT vs 1.35 on DTIC/IFN). This difference was statistically significant ($P < 0.001$), even when adjusted for the time needed to administer chemotherapy ($P < 0.001$).

### DISCUSSION

To date, the pattern of development of combination therapies in melanoma has been for promising results in small scale studies, often at single institutions, which have not then been borne out in larger or multi-centre trials (Nathanson et al, 1981; Luikart et al, 1984; National Cancer Institute of Canada Melanoma Group, 1984). Thus, there is little randomized controlled trial data to support the use of combination chemotherapy, and so far non-randomized data have suggested that although response rates may be enhanced overall survival is not improved (Lakhani et al, 1990; Mulder et al, 1994).

This study was conceived after reports of promising results with the DBCT regimen in advanced melanoma in non-randomized phase II studies (del Prete et al, 1984; McClay et al, 1992). For the trial we adhered to the original DBCT regimen, as reported by del Prete. Other groups have loaded patients with tamoxifen, or spread treatments over 4-week cycles, but evidence for the superiority of these changes over the original schedule is lacking. When the trial was designed there were promising reports of the efficacy of dacarbazine combined with IFN (Falkson et al, 1991) and we elected to use this as our standard therapy. Since then other groups have reported on the utility of dacarbazine and interferon treatment, showing no benefit in terms of response rate or survival over dacarbazine alone (Bajetta et al, 1994; Falkson et al, 1998). With these results in mind, although this study compared DBCT with dacarbazine and IFN, we conclude that standard therapy for advanced melanoma remains single-agent dacarbazine. This conclusion is confirmed by the recent results from the Eastern Co-operative Oncology Group trial comparing DBCT with dacarbazine, which also found no enhancement in response rate or median survival with combination therapy (Chapman et al, 1999).

The response rate and overall survival with the standard therapy used in this study are in keeping with those reported elsewhere, be they for DTIC/IFN or dacarbazine alone (Balch et al, 1995). We found DBCT to be more toxic than DTIC/IFN but tolerable, with few episodes of neutropenic fever or systemic infection. Additionally, DBCT treatment necessitated more time in hospital, an important consideration in a disease where time left to the patients is short. The toxicity observed agrees with previous experience of the regimen: for example, we observed grade IV thrombocytopenia in 43% of patients, which is comparable with the 44% seen in the National Cancer Institute of Canada study and the 39% seen by the Southwest Oncology Group (del Prete et al, 1984; Rusthoven et al, 1996). McClay and Johnston reported fewer adverse haematological effects, but in these studies treatments were administered 4-weekly, and dose reductions were permitted (McClay et al, 1992; Johnston et al, 1997).

Despite early studies with DBCT reporting response rates in excess of 50% more recent non-randomized trials, recruiting greater numbers of patients, have suggested that the true rate in unselected patients is around 15–30% (Rusthoven et al, 1996; Johnston et al, 1997; Margolin et al, 1998). Furthermore, no study to date has shown a survival advantage with this regimen over dacarbazine alone or with IFN. The 26% overall response rate in our study is higher than that seen with DTIC/IFN and similar to the 30% reported in the largest published series to date (Rusthoven et al, 1996). The difference between treatment arms was not statistically significant and did not result in an improvement in median or 1-year survival. Indeed, median response duration was somewhat shorter with DBCT than with DTIC/IFN. It should be noted that a higher proportion than usual (four of nine) of the patients responding to dacarbazine and IFN had complete responses. It could be argued that a potential difference in survival between the treatment arms was masked by patients in the standard arm of the trial receiving cisplatin and tamoxifen after progression. However, less than one-third of the patients assigned to this arm went on to receive further chemotherapy at the time of disease progression, and none of them responded to second-line treatment. Furthermore, the overall survival in the DBCT-treated group is similar to that quoted in the literature for patients receiving single-agent therapies.

Little has changed in the chemotherapeutic management of advanced melanoma in the last few years. The standard of care remains single-agent dacarbazine, with the role of biochemotherapy yet to be determined. Several phase II studies have shown impressive response rates and a number of long-term remissions with combinations of chemotherapy, interleukin-2 (IL-2) and IFN (Richards et al, 1992; Legha et al, 1996; Atkins, 1997; Schultz et al, 1997; Thomson et al, 1997). However, these treatments are toxic and complex to deliver, making them unsuitable for some patients. In any event these results must be treated with caution until confirmed in randomized phase III trials. To date there has been only one published comparison of multi-agent chemotherapy with biochemotherapy. In this IL-2 and IFN improved upon the response rate observed with cisplatin, dacarbazine and tamoxifen, but resulted in poorer median survival (Rosenberg et al, 1999). Neither result achieved statistical significance, although the latter observation brought about early closure of the trial. There are also randomized phase II data to suggest that the addition of subcutaneous IL-2 and IFN-$\alpha$ does not enhance the response rate or median survival of DBCT-treated melanoma patients (Johnson et al, 1998). If, despite these results, the utility of this approach can be established – and there are a number of trials in progress – then the requirement for multiple chemotherapeutic agents within biochemotherapy regimens will need to be addressed (Keilholtz et al, 1998).

In conclusion, this study provides no evidence to support the use of DBCT as standard therapy in advanced melanoma, having yielded no significant improvement in response rate or survival compared with dacarbazine and IFN. It would seem inappropriate to use the four-drug regimen as the control arm in studies of chemotherapy in combination with biological response modifiers, notwithstanding any putative interaction between cisplatin and the latter (Atkins, 1997). Taken in conjunction with the results from the recent large cooperative group study (Chapman et al, 1999), our findings suggest that the standard of care for advanced metastatic melanoma remains single-agent dacarbazine.

© 2000 Cancer Research Campaign

### REFERENCES

Atkins M (1997) The treatment of metastatic melanoma with chemotherapy and biologics. *Curr Opin Oncol* **9:** 205–213

Balch CM, Reintgen DS and Kirkwood JM (1997) Cutaneous melanoma. In: *Cancer: Principles and Practice of Oncology*, 5th edn.), De Vita VT Jr, Hellman S and Rosenberg SA (eds), pp. 1947–1994. Lippincott-Raven: Philadelphia

Bajetta E, Di-Leo A, Zampino MG, Sertoli MR, Comella G, Barduagni M, Giannotti B, Queriolo P, Tribbia G and Bernengo MG (1994) Multicenter randomized trial of dacarbazine alone or in combination with two different doses and schedules of interferon alpha-2a in the treatment of advanced melanoma. *J Clin Oncol* **12:** 806–812

Chapman PB, Einhorn LH, Meyers ML, Saxman S, Destro AN, Panageas KS, Begg CB, Agarwala SS, Schuchter LM, Ernstoff MS, Houghton AH and Kirkwood JM (1999) Phase III multicenter randomized trial of the Dartmouth regimen versus dacarbazine in patients with metastatic melanoma. *J Clin Oncol* **17**: 2745–2752

del Prete SA, Maurer LH, O'Donnell J, Forcier RJ and LeMarbre P (1984). Combination chemotherapy with cisplatin, carmustine, dacarbazine and tamoxifen in metastatic melanoma. *Cancer Treat Rep* **68**: 1403–1405

Falkson CI, Falkson G and Falkson HC (1991) Improved results with the addition of interferon alpha-2b to dacarbazine in the treatment of patients with metastatic malignant melanoma. *J Clin Oncol* **9**: 1403–1410

Falkson CI, Ibrahim J, Kirkwood JM, Coates AS, Atkins MB and Blum RH (1998) Phase III trial of dacarbazine versus dacarbazine with interferon α-2b versus dacarbazine with tamoxifen versus dacarbazine with interferon α-2b and tamoxifen in patients with metastatic malignant melanoma: an Eastern Cooperative Oncology Group study. *J Clin Oncol* **16**: 1743–1751

Johnston SR, Costenla DO, Moore J, Atkinson H, Ahern RP, Dadian G, Riches PG and Gore ME (1998) Randomized phase II trial of BCDT [carmustine (BCNU), cisplatin, dacarbazine (DTIC) and tamoxifen] with or without interferon alpha (IFN-alpha) and interleukin (IL-2) in patients with metastatic melanoma. *Br J Cancer* **77**: 1280–1286

Keilholz U, Conradt C, Legha S, Khayat D, Scheibenbogen C, Thatcher N, Goey SH, Gore M, Dorval T, Hancock B, Punt CJA, Dummer R, Avril MF, Brocker EB, Benhammouda A, Eggermont AA and Pritsch M (1998) Results of interleukin-2-based treatment in advanced melanoma: a case record-based analysis of 631 patients. *J Clin Oncol* **16**: 2921–2929

Lakhani S, Selby P, Bliss JM, Perren TJ, Gore ME and McElwain TJ (1990) Chemotherapy for malignant melanoma: combinations and high doses produce more responses without survival benefit. *Br J Cancer* **61**: 330–334

Lee SM, Betticher DC and Thatcher N (1995) Melanoma: chemotherapy. *Br Med Bull* **51**: 609–630

Legha SS, Ring S, Bedikian A, Plager C, Eton O, Buzaid AC and Papadopoulos N (1996) Treatment of metastatic melanoma with combined chemotherapy containing cisplatin, vinblastine and dacarbazine (CVD) and biotherapy using interleukin-2 and interferon-α. *Ann Oncol* **7**: 827–836

Luikart SD, Kennealy GT and Kirkwood JM (1984) Randomized phase III trial of vinblastine, bleomycin and cis-dichlorodiammine-platinum versus dacarbazine in malignant melanoma. *J Clin Oncol* **2**: 164–168

McClay EF, Mastrangelo MJ, Berd D and Bellet RE (1992) Effective combination of chemo/hormonal therapy for malignant melanoma: experience with three consecutive trials. *Int J Cancer* **50**: 553–556

Margolin KA, Liu PY, Flaherty LE, Sosman JA, Walker MJ, Smith JW III, Fletcher WS, Weiss GR, Unger JM and Sondak VK (1998) Phase II study of carmustine, dacarbazine, cisplatin and tamoxifen in advanced melanoma: a Southwest Oncology Group study. *J Clin Oncol* **16**: 664–669

Mulder NH, van der Graaf WTA, Willemse PHB, Schraffordt Koops H, de Vries EGE and Sleijfer DT (1994) Dacarbazine (DTIC)-based chemotherapy or chemoimmunotherapy of patients with disseminated malignant melanoma. *Br J Cancer* **70**: 681–683

Nathanson L, Kaufman SD and Carey RW (1981) Vinblastine infusion, bleomycin and cis-dichlorodiammine-platinum chemotherapy in metastatic melanoma. *Cancer* **48**: 1290–1294

National Cancer Institute of Canada Melanoma Group (1984) Vinblastine, bleomycin and cis-platinum for the treatment of metastatic malignant melanoma. *J Clin Oncol* **2**: 131–134

Richards J, Mehta N, Ramming K and Skosey P (1992). Sequential chemoimmunotherapy in the treatment of metastatic melanoma. *J Clin Oncol* **10**: 1338–1342

Rosenberg SA, Yang JC, Schwartzentruber DJ, Hwu P, Mmarincola FM, Topalian SL, Seipp CA, Einhorn JH, White DE and Steinberg SM (1999) Prospective randomized trial of the treatment of metastatic melanoma patients using chemotherapy with cisplatin, dacarbazine and tamoxifen alone or in combination with interleukin-2 and interferon alpha-2b. *J Clin Oncol* **17**: 968–973

Rusthoven JJ, Quirt IC, Iscoe, McCulloch PB, James KW, Lohmann RC, Jensen J, Burdette-Radoux S, Bodurtha AJ, Silver HKB, Verma S, Armitage GR, Zee B and Bennett K (1996) Randomized, double-blind, placebo-controlled trial comparing response rates of carmustine, dacarbazine and cisplatin with and without tamoxifen in patients with metastatic melanoma. *J Clin Oncol* **14**: 2083–2090

Schultz MZ, Buzaid AC and Poo WJ (1997) A phase II study of interferon-alpha 2b with dacarbazine, carmustine, cisplatin and tamoxifen in metastatic melanoma. *Melanoma Res* **7**: 147–151

Thompson JA, Gold PJ and Fefer A (1997). Outpatient chemoimmunotherapy for the treatment of metastatic melanoma. *Semin Oncol* **24**: S44–S48

*© 2000 Cancer Research Campaign*
